# Obsessive-compulsive disorder and smartphone addiction among public sector employees in Iran

**DOI:** 10.1186/s40359-025-03913-4

**Published:** 2026-01-03

**Authors:** Ahmad Pirani, Milad Dodangeh, Nastaran Nasirpour, Behrooz Ghanbari, Ahmad Hajebi, Abbas Motevalian

**Affiliations:** 1 Mental Health Research Center, Psychosocial Health Research Institute, Iran University of Medical Sciences, Tehran, Iran; 2 School of Medicine, Iran University of Medical Sciences, Tehran, Iran; 3 Department of Epidemiology, School of Public Health, Iran University of Medical Sciences, Tehran, Iran; 4 Trauma and Injury Research Center, Iran University of Medical Sciences, Tehran, Iran; 5 Research Center for Addiction & Risky Behaviors (ReCARB), Psychosocial Health Research Institute, Iran University of Medical Sciences, Tehran, Iran; 6 Preventive Medicine and Public Health Research Center, Psychosocial Health Research Institute, Iran University of Medical Sciences, Tehran, Iran

**Keywords:** Obsessive-compulsive disorder, Smartphone addiction, Problematic smartphone use

## Abstract

**Background:**

With the ever-increasing use of smartphones, the reciprocal impact of mental disorders and smartphone overuse has become a novel challenge. This study investigated the association between obsessive-compulsive disorder (OCD) and smartphone addiction (SA) in a large sample of Iranian public sector employees.

**Methods:**

This cross-sectional analysis was conducted based on the baseline data of the Employees’ Health Cohort Study of Iran (EHCSIR). The 33-item Smartphone Addiction Scale (SAS) was administered to measure smartphone addiction. The Persian version of the Composite International Diagnostic Interview (CIDI 2.1) was used to identify lifetime OCD in the baseline assessment. Multiple regression analyses were performed to assess the association of OCD with SA and the six dimensions of SAS after adjusting for sociodemographic and job-related variables.

**Results:**

Of the 3,945 participants (mean age = 42.9 years, female = 64.3%), 438 subjects (11.1%) were smartphone-addicted. The prevalence of lifetime OCD was 2.5%. Lifetime OCD was significantly associated with smartphone addiction (OR = 2.27, 95% CI = 1.44–3.58). In addition, lifetime OCD was associated with five dimensions of SAS: daily life disturbance (p-value < 0.05), positive anticipation (*p* < .05), withdrawal (*p* < .01), cyberspace-oriented relationship (*p* < .01), and overuse (*p* < .01).

**Conclusions:**

OCD was associated with a higher likelihood of smartphone addiction and several dimensions of problematic smartphone use. These findings highlight the relevance of psychiatric disorders in shaping digital behavior and may inform interventions targeting mental health in the workplace. Given the cross-sectional design and reliance on self-reported data, causality cannot be inferred and reporting bias may exist. Future studies incorporating objective usage measures and considering additional psychological factors (e.g., stress, impulsivity) are warranted.

## Background

Nowadays, smartphones with various functions, such as communication and entertainment, have become indispensable to every person’s life. Until September 2023, 6.92 billion active smartphone users were reported worldwide [1]. In addition, Iran ranks thirteenth among countries with the highest usage of smartphones, with more than 52 million users in 2021 [2]. As smartphones have increased, so have their additional capabilities and online spaces such as social media, and people have begun to spend more time with them [1, 2]. In the United States, the average person spends three and a half hours on their smartphone daily [3]. This widespread adoption of smartphones has garnered heightened attention toward the reciprocal impact of their problematic use and mental well-being.

A 2024 scoping review of 46 studies reported that individuals with diagnosed psychiatric disorders show elevated rates of problematic Internet use, with the highest frequencies observed in mood, neurodevelopmental, and obsessive–compulsive disorders. Smartphone addiction was identified as one of the most frequently endorsed forms of problematic use [4]. Problematic smartphone use (PSU), also referred to as smartphone addiction (SA) by many scholars, is characterized by a loss of control of smartphone use, leading to impairment of everyday functioning [5]. Compulsive usage, tolerance for more extended and intensive use, withdrawal symptoms from smartphones, and impairment in significant areas of functioning are the four primary elements of SA [6]. A recent systematic review estimated that the prevalence of SA around the world is approximately 27% [7]. Previous studies suggest the possible relationships between SA and physical and psychological disorders such as musculoskeletal pain, sleep disorders, anxiety, depression, and suicide [8–11]. A significant point of contention in this subject concerns the connection between obsessive-compulsive disorder (OCD) and SA.

OCD is a condition in which an unwanted and persistent thought is followed by compulsive behavior, such as washing hands frequently, checking frequently, and hoarding to avoid this unpleasant idea [12, 13]. A limited number of studies have addressed the correlation between SA and OCD [13–15]. A study with 550 participants in the US revealed a potential relationship between smartphone overuse and obsessive-compulsive symptoms [14]. Another study conducted on 934 college students in Italy showed that users with PSU had higher levels of OCD symptoms [16]. Lee et al., in a study on 755 adults, also noted that SA could be associated with OCD [17].

Nonetheless, the information currently available in this area is based on research with restricted demographic groups, such as adolescents and college students, or small samples, with insufficient consideration of the confounding factors that may not be indicative of the broader population. Therefore, to fill the gaps in this field, this study aimed to investigate the association between OCD and addiction to smartphone usage in a larger population.

The causal direction between OCD and SA remains unclear. Some evidence suggests that OCD symptoms, particularly compulsive checking and reassurance-seeking behaviors, may predispose individuals to excessive smartphone use, providing a cognitive–behavioral mechanism for the link [18, 19]. Conversely, the design of smartphones and their notification systems may reinforce compulsive patterns of checking and gradually intensify obsessive tendencies, suggesting that SA may exacerbate or even trigger OCD-like symptoms [20–22]. While both directions are plausible, framing SA as a consequence of OCD is theoretically more consistent with models of addiction as a compulsive behavior [23].

Furthermore, cultural context may shape how SA manifests. In Iran, where smartphone penetration is among the highest in the region, devices are widely used not only for communication but also for professional, educational, and social purposes. Social norms, including the central role of online messaging platforms in daily interactions, may encourage continuous connectivity, which can reinforce compulsive patterns of use. Understanding these cultural influences is essential when interpreting the relationship between OCD and smartphone use in Iranian populations.

Based on this premise, we examined the association between OCD and SA, as well as the six dimensions of the Smartphone Addiction Scale (SAS), using baseline data from the Employees’ Health Cohort Study of Iran (EHCSIR).

## Methods

### Study design

This study is a cross-sectional analysis utilizing baseline data from the Employees Health Cohort Study of Iran (EHCSIR), a prospective cohort study designed to explore various occupational and non-occupational factors influencing non-communicable diseases among public sector employees in Iran. The reference population of EHCSIR included more than 15,000 employees working in 43 different units within Tehran province, primarily in schools, hospitals, health centers associated with the Iran University of Medical Sciences, and the Ministry of Health and Medical Education’s headquarters staff. All active, non-temporary employees were eligible for the study. Initially targeting to enroll 10,000 employees, recruitment efforts were curtailed in March 2020 due to the implementation of social distancing measures prompted by the COVID-19 pandemic. Between July 2017 and March 2020, the study successfully recruited 4,886 employees.

### Baseline assessments

Each of the 4,886 participants completed an extensive baseline assessment, which took approximately 7 h at a single facility. This assessment included multiple face-to-face interviews to gather medical, psychological, nutritional, and sociodemographic information. Participants also underwent various clinical examinations such as blood pressure measurements, electrocardiograms, pulmonary function tests, and auditory and visual assessments. Additionally, anthropometric data were collected along with biospecimens (blood and urine) for laboratory analysis. Participants also filled out a self-administered questionnaire on a tablet.

### Measurements

#### Smartphone addiction

Participants in the EHCSIR cohort were assessed for smartphone addiction while at the cohort center completing their baseline assessments. The assessment tool used was the Persian version of the Smartphone Addiction Scale (SAS), a 33-item, self-administered questionnaire presented on a tablet. This scale employs a six-point Likert scale, ranging from ‘1 = strongly disagree’ to ‘6 = strongly agree,’ and includes six subscales: daily life disturbance, positive anticipation, withdrawal symptoms, cyberspace-oriented relationship, overuse, and tolerance. The scores from each subscale are aggregated, with higher scores indicating a greater likelihood of smartphone addiction. The validity and reliability of the Persian version of the SAS were confirmed in a study conducted by Kheradmand et al. on a sample of university students. The scale demonstrated excellent internal consistency (Cronbach’s alpha = 0.93), high test–retest reliability (intraclass correlation = 0.996), strong concurrent validity with the Persian Internet Addiction Test (*r* =.70), and good construct validity (KMO = 0.92; Bartlett’s test significant). The authors also reported an optimal cutoff score of 106 with sensitivity of 80% and specificity of 86% [24].

#### Obsessive-compulsive disorder

The Composite International Diagnostic Interview version 2.1 (CIDI 2.1) represents a widely recognized and thorough instrument utilized in structured interviews aimed at identifying psychiatric conditions, including obsessive-compulsive disorder (OCD). Developed by the World Health Organization (WHO), the CIDI 2.1 draws upon the International Classification of Diseases, Tenth Revision (ICD-10), and the Diagnostic and Statistical Manual of Mental Disorders, 4th Edition (DSM-IV), specifically tailored for epidemiological investigations within the general populace [25]. Notably, this tool can be effectively administered by individuals lacking prior clinical training and boasts a concise completion time [25].

Extensive research has demonstrated the robust validity and reliability of the CIDI for most diagnostic sections across various contexts and languages [26, 27]. Notably, the Persian version of CIDI 2.1 has exhibited acceptable psychometric properties [28, 29].

In the EHCSIR project, four trained psychologists employed the lifetime Persian version of CIDI 2.1 to evaluate common mental disorders among EHCSIR participants during the initial assessment. Subsequently, the presence of OCD was ascertained based on the diagnostic criteria outlined in the DSM-IV.

### Covariate factors

The EHCSIR baseline dataset was utilized for the extraction of socio-demographic variables, encompassing gender, age, marital status, educational attainment, job, and household assets and amenities. The wealth index was calculated using principal component analysis (PCA) based on asset data.

### Ethical statement

This research was reviewed and approved by the Research Ethics Committee of the Iran University of Medica Sciences and Health Services (Ethics approval number IR.IUMS.FMD.REC.1399.240). All the steps of the study were performed by the latest version of the Helsinki Regulation [30]. Written informed consent was obtained from the subjects while the anonymity and confidentiality of their identity were granted.

### Handling missing data

The study sample consisted of 4,886 public sector employees enrolled in the EHCSIR project. Among these, 3,945 participants owned a smartphone and provided complete data on OCD, SAS, and other covariates. Details of the missing data are as follows: 146 participants did not complete the Composite International Diagnostic Interview (CIDI); 433 did not respond to the tablet-based SAS questionnaire; and 216 had incomplete covariate information. Additionally, 154 participants who completed the SAS questionnaire did not own a smartphone.

Given the observations that non-response largely stemmed from the time-consuming nature of basic evaluations and the non-attendance at some assessment stations, which were influenced by day-to-day fluctuations in the number of participants arriving at the study center rather than by any participant characteristic, the missing data were assumed to be missing completely at random (MCAR). Consequently, the primary analyses were conducted on the subset of complete cases. Additionally, to further explore the potential impact of missing data on our study results, a sensitivity analysis was conducted under four distinct scenarios.

### Statistical analysis

Descriptive statistics included the mean and standard deviation (Mean ± SD) for continuous variables, and frequencies (percentages) for categorical variables. Principal component analysis (PCA) was used to derive a wealth index from asset data, which was then categorized into three groups: high, middle, and low status. A binary variable for SA was created with a cutoff point of 106, following the criteria established by prior studies.

Logistic regression analyses were employed to explore the influence of OCD on smartphone addiction. We calculated odds ratios (ORs) and 95% confidence intervals (CIs) to assess the impact of each SAS dimension as well as the total SAS score on SA, controlling for sociodemographic factors. Three models were utilized: an unadjusted model, a minimally adjusted model (Model 1) accounting for gender and age, and a fully adjusted model (Model 2) which also included marital status, educational attainment, employment status, and wealth index. Statistical significance was determined at a p-value of 0.05. All statistical analyses were conducted using STATA version 14.0.

To address the impact of missing data on our study outcomes, we conducted a sensitivity analysis under four scenarios. For OCD diagnosis, multiple imputation was used to fill in missing values among participants who did not complete the CIDI interview (*n* = 146). For smartphone addiction, multiple imputation was applied to the missing SAS data, treating it separately as a binary outcome and as a continuous score for those who did not respond to all of the items of the SAS questionnaire (*n* = 433; most missing only 1–3 of the 33 items). For participants without a smartphone (*n* = 154), we assigned a negative outcome for smartphone addiction, based on the logical assumption that the absence of a smartphone precludes addiction to it.

## Results

Among the 4,886 participants registered in the EHCSIR, 3,945 individuals who owned smartphones and provided a complete data set were included in this analysis. The total score for the Smartphone Addiction Scale (SAS) ranged from 33 to 192, with a mean of 76.1, a median of 75.0, and a standard deviation of 24.2. Furthermore, 438 individuals (11.1%) met the criteria for smartphone addiction (SA). The characteristics of the study population according to SA are delineated in Table 1. The majority of the participants were female (64.3%). The mean age of the participants was 42.9 years (SD = 8.1), with an age range from 23 to 84 years. Notably, participants who were aged 34 years or younger exhibited a higher prevalence of smartphone addiction (*p* <.001) compared to other age groups. Furthermore, it was found that 3.1% of the respondents had a lifetime OCD. Individuals who suffered from OCD had a higher prevalence of smartphone addiction (*p* <.001). Although lifetime OCD prevalence in our sample was low, which may limit the precision of some estimates, the relatively large number of cases (*n* = 122) compared with the overall sample (*n* = 3,823 non-OCD participants) provides sufficient power to detect associations and enhances the stability of our main findings.

**Table 1:** Characteristics of study subjects by smartphone addiction status in the EHCSIR

		*N*	Smartphone addiction		
			*n*	%	*P*-value
Gender	Male Female	1409 2536	168 287	11.9 11.3	0.57
Age	34 ≥ 35–44 45–54 ≥ 55	650 1762 1234 299	106 197 125 27	16.3 11.2 10.1 9.0	< 0.001*
Marital status	Married Never married Previously married	3187 555 203	330 97 28	10.4 17.5 13.8	< 0.001*
Education	Diploma ≥ Associate-Bachelor’s level Master’s level ≤	1058 1919 968	101 229 125	9.6 11.9 12.9	< 0.05*
Job	Office work Academic Health Professional Service work	1334 118 1839 654	132 18 234 71	9.9 15.3 12.7 10.9	< 0.05*
Wealth index	Low Medium High	937 2035 973	103 238 114	11.0 11.7 11.7	0.84
Lifetime OCD	No Yes	3823 122	429 26	11.2 21.3	< 0.001*

^*^Significant

As detailed in Table 2, the results of logistic regression analyses revealed that lifetime OCD was significantly associated with SA (OR = 2.27, CI95%: 1.44–3.58). The likelihood of SA was considerably higher in male gender (OR = 0.76), younger age group (≤ 34 years old vs. ≥55 years old, OR = 1.83), never married (OR = 1.78), and previously married (OR = 1.72) individuals.

**Table 2:** Unadjusted and adjusted odds ratios for smartphone addiction based on sociodemographic characteristics and lifetime OCD

		Unadjusted OR		Adjusted OR^*^	
		Point estimate	95% CI	Point estimate	95% CI
Gender	Female	0.94	0.77–1.15	0.76	0.60–0.96
	Male	1.00		1.00	
Age group	≤ 34	1.96	1.26–3.07	1.83	1.15–2.93
	35–44	1.27	0.83–1.93	1.23	0.79–1.90
	45–54	1.14	0.73–1.76	1.10	0.71–1.72
	≥ 55	1.00		1.00	
Marital status	Married	1.00		1.00	
	Never Married	1.83	1.43–2.35	1.78	1.37–2.31
	Previously married	1.39	0.91–2.10	1.72	1.11–2.66
Educational attainment	Diploma ≥	1.00		1.00	
	Associate-Bachelor’s level	1.28	0.98–1.66	1.23	0.91–1.67
	Master’s level ≤	1.47	1.10–1.96	1.34	0.94–1.92
Job	Office work	1.00		1.00	
	Academic	1.64	0.96–2.79	1.43	0.73–2.83
	Health Professional	1.33	1.06–1.66	1.30	1.03–1.64
	Service work	1.11	0.82–1.50	1.10	0.74–1.62
Wealth index	Low	1.00		1.00	
	Medium	1.07	0.84–1.37	1.02	0.78–1.33
	High	1.07	0.81–1.43	1.07	0.77–1.48
Lifetime OCD	No	1.00		1.00	
	Yes	2.14	1.37–3.34	2.27	1.44–3.58

^*^Adjusted for gender, age, marital status, educational attainment, job, and wealth index

Our study findings demonstrate a strong relationship between lifetime OCD and SAS scores. In addition to the total SAS score, lifetime OCD exhibited significant associations with five out of six smartphone addiction subscales after controlling for covariates. These include daily life disturbance (*P* <.05), positive anticipation (*P* <.05), withdrawal symptoms (*P* <.01), cyberspace-oriented relationships (*P* <.01), and overuse (*P* <.01), as detailed in Table 3.

**Table 3:** Association between lifetime OCD and smartphone addiction subscales

SAS subscales	Unadjusted		Model 1		Model 2	
	ß	*P*-value	ß	*P*-value	ß	*P*-value
1.Daily life disturbance	0.22	< 0.05	0.22	< 0.05	0.21	< 0.05
2.Positive anticipation	0.19	< 0.05	0.17	< 0.05	0.20	< 0.05
3.Withdrawal	0.29	< 0.01	0.28	< 0.01	0.30	< 0.01
4.Cyberspace-oriented relationship	0.25	< 0.01	0.23	< 0.01	0.26	< 0.01
5.Overuse	0.28	< 0.01	0.24	< 0.05	0.30	< 0.01
6.Tolerance	0.18	0.05	0.17	0.06	0.17	0.07
Total SAS	7.71	< 0.01	7.19	< 0.01	8.00	< 0.01

Model 1: Adjusted for gender and age

Model 2: Adjusted for gender, age, marital status, educational attainment, job, and wealth index

Sensitivity analyses confirmed the robustness of the association between lifetime OCD and smartphone addiction (Table 4). Using multiple imputation for missing exposure data, lifetime OCD remained significantly associated with both smartphone addiction (adjusted OR = 2.31, 95% CI = 1.48–3.61) and higher total SAS scores (β = 6.42, 95% CI = 1.79–11.04). Similar results were obtained when imputing missing outcome data (adjusted OR = 2.13, 95% CI = 1.34–3.38; β = 7.55, 95% CI = 3.23–11.87). Finally, when participants without smartphones were classified as not addicted, the association persisted (adjusted OR = 2.25, 95% CI = 1.42–3.58; β = 6.63, 95% CI = 1.89–11.37). Across all models, the direction and magnitude of the effect estimates were consistent with the main analyses.

**Table 4:** Sensitivity analyses of the association between lifetime OCD and smartphone addiction

Multiple imputation of exposure*							
Exposure		Smartphone addiction (*n* = 4088)			Total SAS (*n* = 4088)		
		**OR**	**95% CI**	**P value**	ß	**95% CI**	**P value**
**Lifetime OCD**							
	Unadjusted	2.33	1.52–3.58	< 0.001	7.35	2.63–12.08	< 0.005
	Model 1	2.25	1.47–3.45	< 0.001	6.02	1.56–10.85	< 0.01
	Model 2	2.31	1.48–3.61	< 0.001	6.42	1.79–11.04	< 0.01
Multiple imputation of outcome**							
**Exposure**		**Smartphone addiction** (*n*** = 4378)**			**Total SAS** (*n*** = 4378)**		
		**OR**	**95% CI**	**P value**	ß	**95% CI**	**P value**
**Lifetime OCD**							
	Unadjusted	2.00	1.27–3.15	< 0.001	7.22	2.87–11.58	< 0.001
	Model 1	1.94	1.23–3.06	< 0.001	6.76	2.42–11.10	< 0.005
	Model 2	2.13	1.34–3.38	< 0.001	7.55	3.23–11.87	< 0.001
**Not having smartphone considered as no smartphone addiction**							
**Exposure**		**Smartphone addiction** (*n*** = 4099)**			**Total SAS** (*n*** = 4099)**		
		**OR**	**95% CI**	**P value**	ß	**95% CI**	**P value**
**Lifetime OCD**							
	Unadjusted	2.12	1.35–3.35	< 0.001	7.17	2.27–12.06	< 0.005
	Model 1	2.03	1.28–3.21	< 0.005	5.90	1.08–12.73.08.73	< 0.05
	Model 2	2.25	1.42–3.58	< 0.001	6.63	1.89–11.37	< 0.01

Model 1: Adjusted for gender and age

Model 2: Adjusted for gender, age, marital status, education attainment, job, and wealth index

*For individuals who did not complete the CIDI interview during baseline assessments, data on lifetime OCD exposure were imputed using the mi command in STATA

**For study participants who did not complete the tablet-based, self-administered SAS questionnaire, outcomes related to smartphone addiction (both the binary outcome and the total SAS score) were imputed using a multiple imputation technique

## Discussion

The present study identified significant associations between OCD and both the binary variable SA and five out of six SAS subscales: daily life disturbance, positive anticipation, withdrawal, cyberspace-oriented relationships, and overuse, in the Employees’ Health Cohort Study of Iran.

Consistent with our findings, Wickord et al., reported in a survey of 399 cases in Germany that OCD had a predictive effect on PSU. They attributed this finding to the fact that PSU acts as a maladaptive coping mechanism to distract users from unpleasant feelings [20]. Aroyewun et al. also found that OCD was significantly predictive for SA [21]. However, the results from other previous studies have not been conclusive in this field. For instance, Nahas et al. failed to achieve an association between PSU and OCD among Lebanese adults due to the inadequate number of participants, suggesting a study with a larger sample size [31]. Moreover, in a study investigating psychiatric risk factors for PSU, obsessive-compulsive symptoms were not found to be a significant predictor of PSU [32].

It is important to note that these associations do not imply causality, as the cross-sectional design precludes determining temporal direction. While some longitudinal studies suggest that excessive smartphone use may exacerbate obsessive-compulsive symptoms, others indicate that compulsivity inherent in OCD may predispose individuals to problematic smartphone use. Thus, a bidirectional relationship remains plausible and warrants further longitudinal investigation. Although numerous research has explored the relationship between addiction to smartphones and mental disorders, little dedicated study can be found on the psychological antecedents of SA. Furthermore, most of these reports include other neurotic disorders, namely anxiety and depression. Pourafshari et al. demonstrated in a study using an online questionnaire that anxiety and depression might result in PSU among Iranian people [33]. Similarly, depression has been identified as a risk factor for SA in Saudi Arabia’s adult population [34]. In a 2021 meta-analysis, Busch et al. concluded that anxiety and depression could lead to PSU. They proposed that anxious and depressed individuals spend more time on smartphones to avoid undesirable emotions, resulting in PSU [5].

Indeed, obsessive thoughts and urges, as defined by the DSM-5-TR for OCD, ultimately result in anxiety or distress in most OCD people [12]. Additionally, one of the predisposing factors for SA is an increase in the level of stress ensued by the loss of self-control, leading to SA [35]. According to this theory, SA is in connection with the fundamental anxiety of OCD. Furthermore, from the phenomenological point of view, by considering compulsion as an inescapable repetitive act to prevent distress and by viewing addiction as a behavior aimed at receiving an instant gratification of urges [12, 36], whether the higher smartphone involvement in OCD individuals relates to the compulsive aspect of their underlying disorder or is an addictive behavior requires further studies. Alternative explanations should also be considered. Stress and anxiety may act as mediators in the OCD–SA relationship, with individuals turning to smartphones as a coping strategy to alleviate distress. Moreover, compulsive checking behaviors characteristic of OCD may extend into digital contexts, reinforcing excessive smartphone use patterns.

Given the ever-increasing use of smartphones and the increase in unusual smartphone-based compulsive behaviors such as frequent checking, hoarding pictures, films, virtual friends, etc., the necessity of a new approach to the symptomatology of OCD seems inevitable. A study on 101 Pinterest users uncovered a potential similarity between physical and digital hoarding [37]. Likewise, Carmi et al. suggested “digital contamination” and “digital symmetry” as unexplored areas of OCD symptoms [15]. In the same way, the significant association of OCD and the subscale of cyberspace-oriented relationships in our study can be seen as one of the symptoms of OCD in the form of hoarding.

On the other hand, from a coping strategy perspective, since one of the consequences of OCD is the reduction of interpersonal and social relationships, we can conclude that cyberspace-oriented relationships and the feeling of empowerment in bringing about virtual relationships can be a kind of compromise for this defect and the person cover his relational problems to some extent. Such an alternative relational space and the feeling of greater control will ultimately lead to satisfaction and functionality. Nevertheless, it should be noted that this extreme presence in virtual spaces is likely to create a vicious cycle that leads to further isolation.

These findings must also be interpreted within the cultural context of Iran, a country with one of the highest smartphone penetration rates globally. Social norms that encourage continuous connectivity, coupled with stigma surrounding mental health treatment, may intensify reliance on digital platforms for coping and social interaction. Such cultural factors may amplify the observed associations between OCD and problematic smartphone use.

### Practical workplace implications and future directions

In practical terms, these results suggest that employers and occupational health services should be aware that employees with OCD may be more susceptible to problematic smartphone use, which could affect concentration, productivity, and workplace interactions. Interventions might include digital wellness programs, confidential support services, and promoting structured work breaks and alternative coping strategies to mitigate compulsive smartphone engagement.

Future research could explore potential mediators such as stress, anxiety, or compulsive checking behaviors that may explain the pathway from OCD to SA. Moderators such as gender, job type, or work environment (e.g., remote vs. on-site work) could also influence these relationships, guiding tailored interventions and workplace policies. Additionally, studies could benefit from Ecological Momentary Assessment (EMA) or smartphone-based tracking to capture real-time patterns of smartphone use and compulsive behaviors in naturalistic settings. EMA would allow researchers to assess temporal associations between OCD symptoms and smartphone engagement, identify contextual triggers, and examine dynamic interactions with mediators such as stress or mood.

Our study, leveraging the EHCSIR database, benefited from a large sample size of Iranian employees with diverse demographic characteristics. The use of the CIDI 2.1 diagnostic questionnaire, superior to symptomatic questionnaires used elsewhere, and the 33-item SAS questionnaire, compared to shorter versions used in other studies, are strengths of our research. Additional limitations should be noted. First, the cross-sectional nature of this study precludes conclusions about causality. Second, our reliance on self-reported measures may introduce recall and social desirability biases. Third, we did not have access to objective smartphone usage data (e.g., screen-time logs), which could provide more precise estimates of problematic use. Finally, although we adjusted for a wide range of sociodemographic and job-related variables, additional psychological confounders such as impulsivity, stress, or generalized anxiety were not included and should be examined in future studies. Further experimental and cohort studies are necessary. Given the cohort database, researchers have the opportunity to longitudinally follow up cases to explore potential bidirectional relationships between these variables. Additionally, considering the varied uses of smartphones for social networking, information gathering, and entertainment, we suggest designing future studies to explore specific types of addiction using tailored questionnaires [38]. This approach may enhance our understanding of how OCD might lead to SA and identify specific smartphone usage patterns prone to addiction in individuals with OCD, and clarify whether these associations differ according to OCD symptom severity.

## Conclusions

In terms of SA, OCD has been less frequently addressed than other neurotic disorders. Our study revealed that OCD was significantly associated with SA, confirming that psychiatric disorders can shape smartphone use patterns. However, due to the cross-sectional nature of the data, causality cannot be inferred. Future longitudinal studies are needed to clarify whether OCD symptoms precede smartphone addiction or whether excessive smartphone use exacerbates obsessive-compulsive tendencies. Clinically, our findings suggest that digital behavior tracking could be used to monitor compulsive tendencies in OCD, while smartphone-based therapeutic applications may represent a promising intervention avenue. Importantly, the study utilized data from a large, well-characterized cohort of Iranian employees, enhancing the robustness and generalizability of the findings. More broadly, recognizing the functional consequences of OCD in the digital age may open new directions for both research and treatment.

## Data Availability

The data analyzed during the current study are available from the corresponding author upon reasonable request.

## Abbreviations

EHCSIR: Employees’ Health Cohort Study in Iran
OCD: Obsessive-Compulsive Disorder
SA: Smartphone Addiction
PSU: Problematic Smartphone Use
SAS: Smartphone Addiction Scale
CIDI 2.1: Composite International Diagnostic Interview version 2.1
WHO: World Health Organization
DSM-IV: Diagnostic and Statistical Manual of Mental Disorders, 4th Edition
ICD-10: International Classification of Diseases, Tenth Revision
APA: American Psychiatric Association
